# Supplementary material for: Backbone-independent NMR resonance assignments of methyl probes in large proteins
Source: Nat Commun. 2021 Jan 29;12:691. doi: 10.1038/s41467-021-20984-0 (PMC7846771; doi:10.1038/s41467-021-20984-0)
Supplement: Supplementary file 1 — Supplementary Information [file 41467_2021_20984_MOESM1_ESM.pdf]

SUPPLEMENTARY INFORMATION

Backbone-independent NMR resonance assignments of methyl probes in large proteins

Santrupti Nerli[1]*, Viviane S. De Paula[2,3,4]*, Andrew C. McShan[2,4] and Nikolaos G. Sgourakis[2,4]

[1]Department of Biomolecular Engineering, University of California, Santa Cruz, CA 95064
[2]Department of Chemistry and Biochemistry, University of California, Santa Cruz, CA 95064
[3]Núcleo Multidisciplinar de Pesquisas, Universidade Federal do Rio de Janeiro, Duque de Caxias, RJ, Brazil, 25245-390
[4]Present address: Department of Pathology and Laboratory Medicine, The Children's Hospital of Philadelphia, and Department of Biochemistry and Biophysics, Perelman School of Medicine, University of Pennsylvania, 3401 Civic Center Blvd, Philadelphia, PA, 19104, USA

*These authors contributed equally to this work.
Correspondence: nikolaos.sgourakis@pennmedicine.upenn.edu

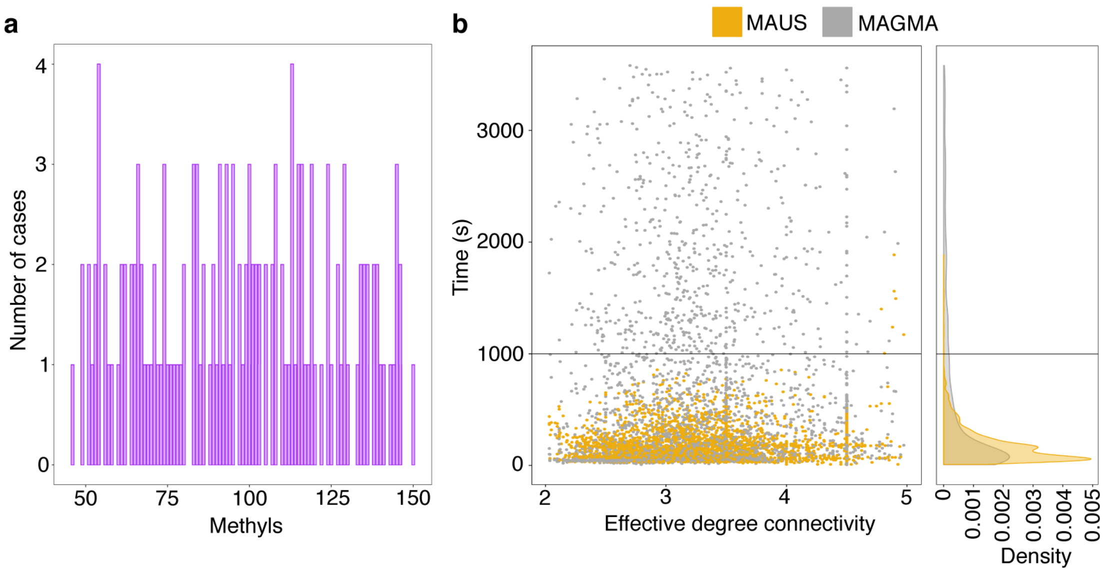

Supplementary Figure 1. Comparison of VF2 and SAT subgraph isomorphism enumeration algorithms using simulated data graphs from the PDB. (a) Bar plot showing the size distribution of 147 representative PDB structures ranging from 50 to 150 methyls (~25-85 kDa), that were used to generate 3,275 simulated methyl assignment inputs (isomorphic structure and data graphs). **(b)** Scatter plot showing the performance (in time, seconds) of the MAUS special-purpose satisfiability algorithm (golden) and MAGMA's VF2 (grey)[1], as a function of effective degree connectivity (EDC), used to estimate the sparsity of the input data graph. EDC is calculated as (2 * number of edges) / (number of nodes), where a node is a methyl-bearing residue and an edge represents an NOE between two residues. Runtime distributions are shown on the right panel. The outputs of both algorithms were identical for all targets. VF2 and SAT runtimes exceed 1,000 seconds (indicated by black horizontal line) for 20% and 0.2% out of 3,275 total simulation cases, respectively.

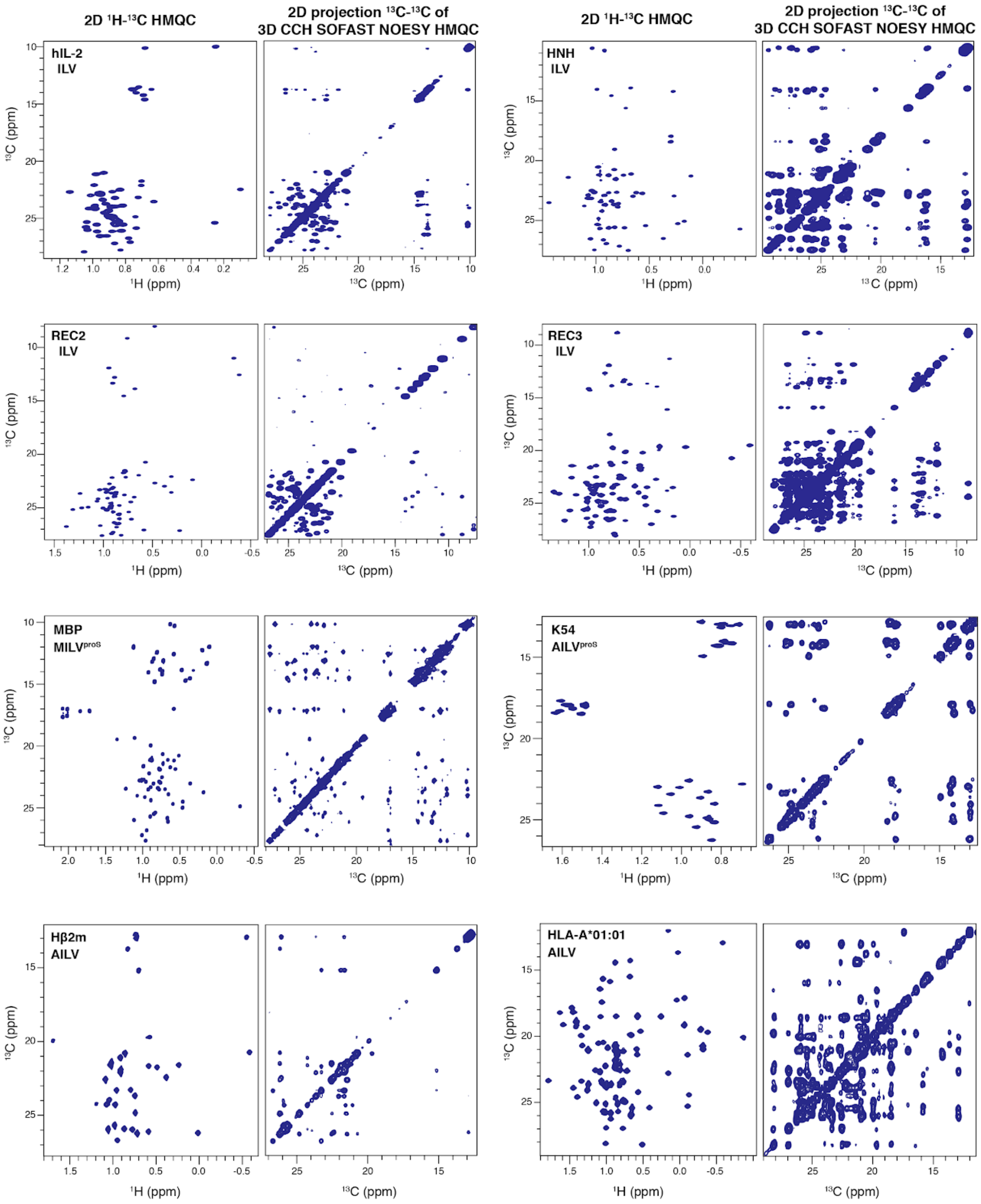

Supplementary Figure 2. Input NMR data provided for MAUS for all benchmark and blind targets in this work. 2D ^{1}H-^{13}C methyl SOFAST HMQC spectra and 2D $^{13}C_M$-$^{13}C_M$ projections of 3D C_M-C_MH_M SOFAST NOESY HMQC (300 ms mixing time) for the HNH, REC2, and REC3 domains of Cas9, IL-2, MBP, K54, β$_2$-microglobulin (Hβ$_2$m) and HLA-A01. The isotopic labeling scheme for each construct is noted. Data acquisition parameters are listed in **Supplementary Table 2**.

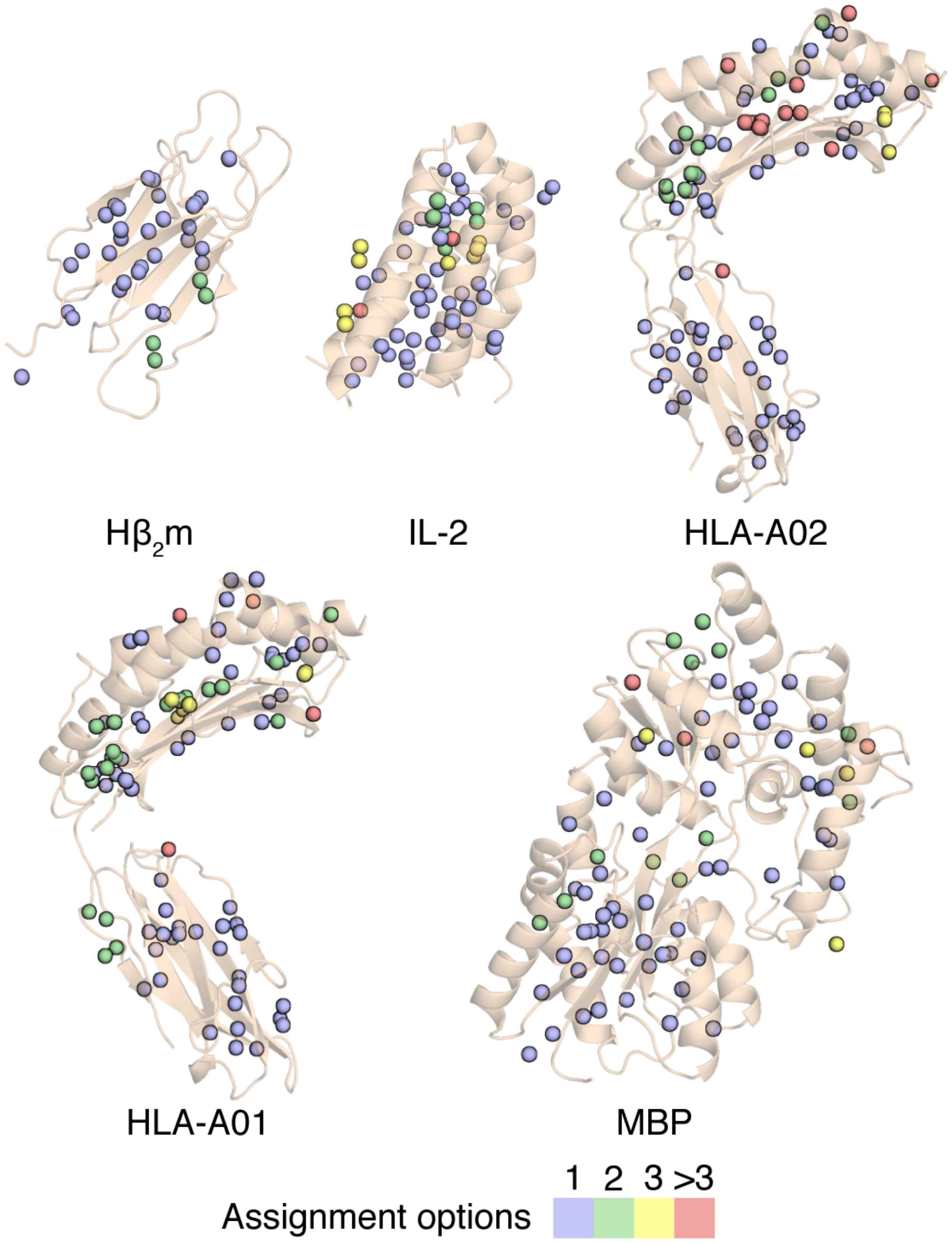

Supplementary Figure 3. Summary of assignment completeness for 5 benchmark targets. Number of valid assignment options produced by MAUS for each methyl-bearing residue, are shown for 5 protein targets of different folds (Hβ_2m, IL-2, HLA-A02, HLA-A01 and MBP). The colored spheres represent valid resonance assignment options, violet, green, yellow and red for 1, 2, 3 and >3 option/s, respectively. Assignment accuracy is 100%, for targets.

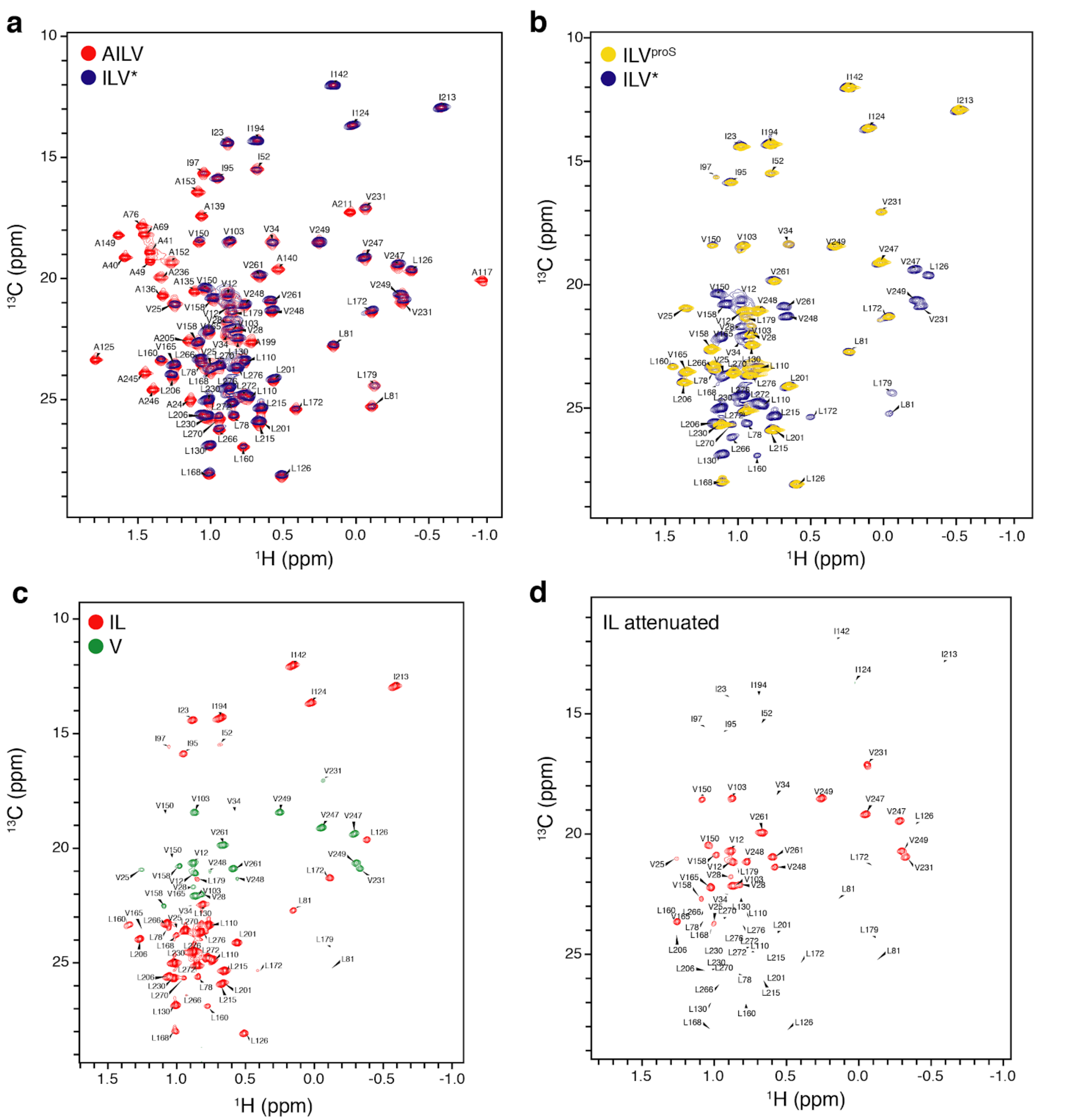

Supplementary Figure 4. Identification of residue types and stereospecificity for the methyl resonances of HLA-A01. (a) Overlay of 2D ^{1}H-^{13}C SOFAST HMQC spectra of AILV (red) and ILV* (blue) labeled HLA-A01. Comparison allows clear identification of Ala methyls. **(b)** Overlay of 2D ^{1}H-^{13}C SOFAST HMQC spectra of ILV^{pros} (yellow) and ILV* (blue) labeled HLA-A01. Comparison allows clear identification of stereospecific Leu δ_1/δ_2 and Val γ_1/γ_2 methyls. **(c)** and **(d)** phase-sensitive 2D ^{1}H-^{13}C Constant-Time HMQC experiments recorded on ILV* labeled HLA-A01. In **(c)** Ile δ_1/Leu δ_1/δ_2 and Val γ_1/γ_2 methyl signals are positive (red) and negative (green), respectively. In **(d)** Ile δ_1/Leu δ_1/δ_2 and Val γ_1/γ_2 methyl signals are attenuated and positive (red), respectively. All experiments were acquired on ~500 μM HLA-A01 sample and recorded at ^{1}H fields of 750 or 800 MHz at 25 ºC.

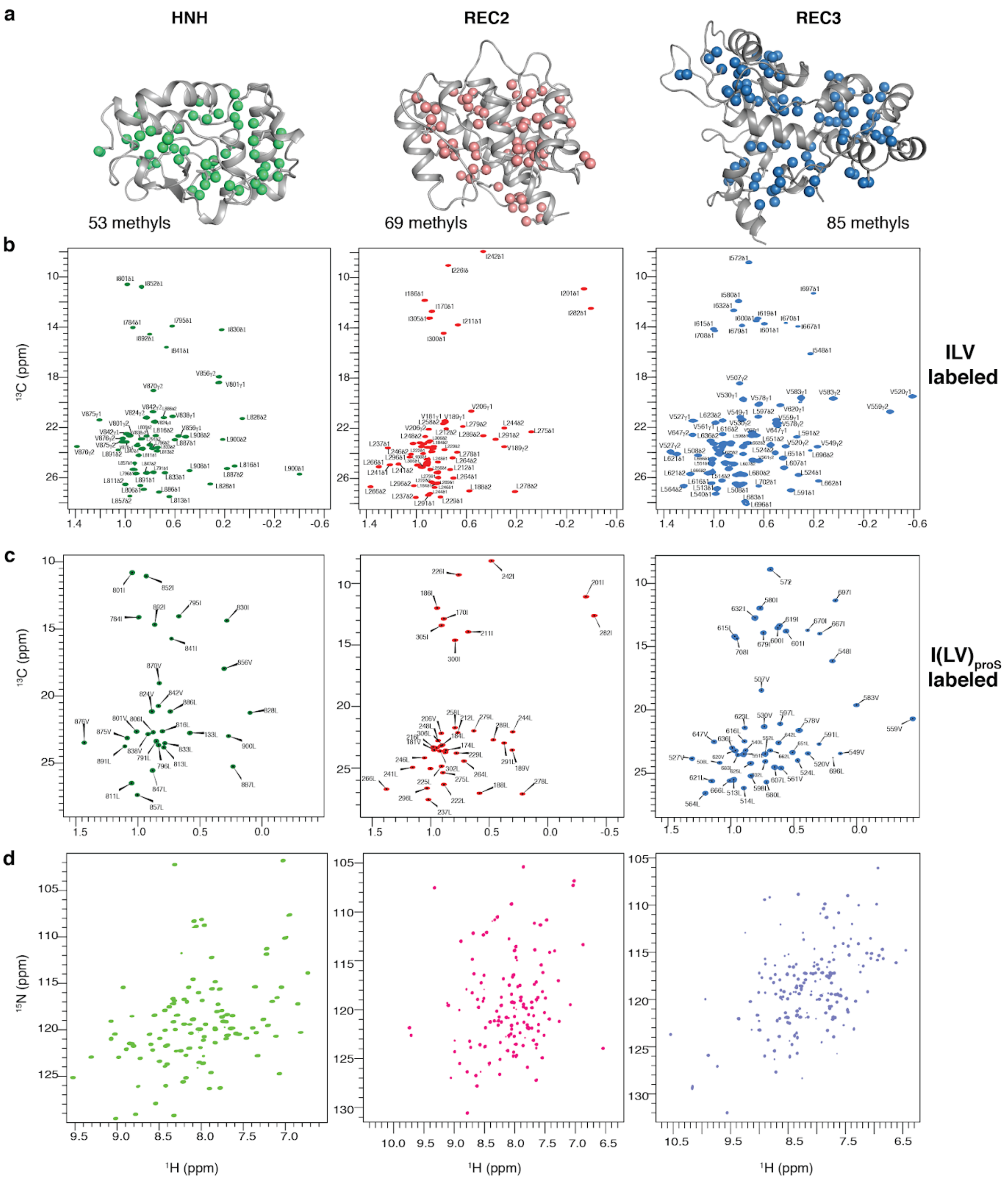

Supplementary Figure 5. Divide-and-conquer resonance assignment strategy for three domains of the Cas9 holoenzyme. (a) Distribution of the ILV methyl groups in the Cas9 domains studied in isolation. The backbone is shown with ribbons, ILV methyl groups as spheres. Color coding: HNH, green; REC2, red; REC3, blue. **(b)** Corresponding ^{1}H-^{13}C methyl HMQC spectra of [U-^{2}H,^{15}N, Ileδ$_1$-$^{13}CH_3$; Leu, Val-$^{13}CH_3$/$^{13}CH_3$], **(c)** ^{1}H-^{13}C methyl HMQC spectra of [U-^{2}H,^{15}N, Ileδ$_1$-$^{13}CH_3$, Leu, Val proS]-labeled HNH, REC2 and

REC3 domains and **(d)** ^{1}H-^{15}N TROSY HSQC acquired at 800 MHz, 25 ºC. Leu/Val methyl groups are labeled with their stereospecific assignments. The methyl chemical shifts of the residues in the hydrophobic core of these building blocks will not be affected much by the disruption of the full-length protein provided that the smaller units fold in a similar manner in isolation and in the full-length Cas9. Backbone chemical shifts (^{15}N, ^{1}HN, $^{13}C\alpha$, $^{13}C\beta$, ^{13}CO) were assigned through a combination of 3D HNCA, 3D HN(CA)CB and 3D HNCO triple-resonance experiments using a sequential assignment method. Stereospecific sidechain methyl chemical shift assignments were obtained from [U-^{2}H,^{15}N, Ileδ_1-$^{13}CH_3$, Leu, Val proS]-labeled samples. Sidechain methyl assignments were assigned and cross-validated by obtaining methyl-to-amide NOEs from 3D H_N-C_MH_M and 3D N-C_MH_M SOFAST NOESY experiments. Iterative completion and validation of the methyl assignments were confirmed through NOEs obtained from 3D H_M-C_MH_M and 3D C_M-C_MH_M SOFAST NOESY experiments (detailed in **Supplementary Results**).

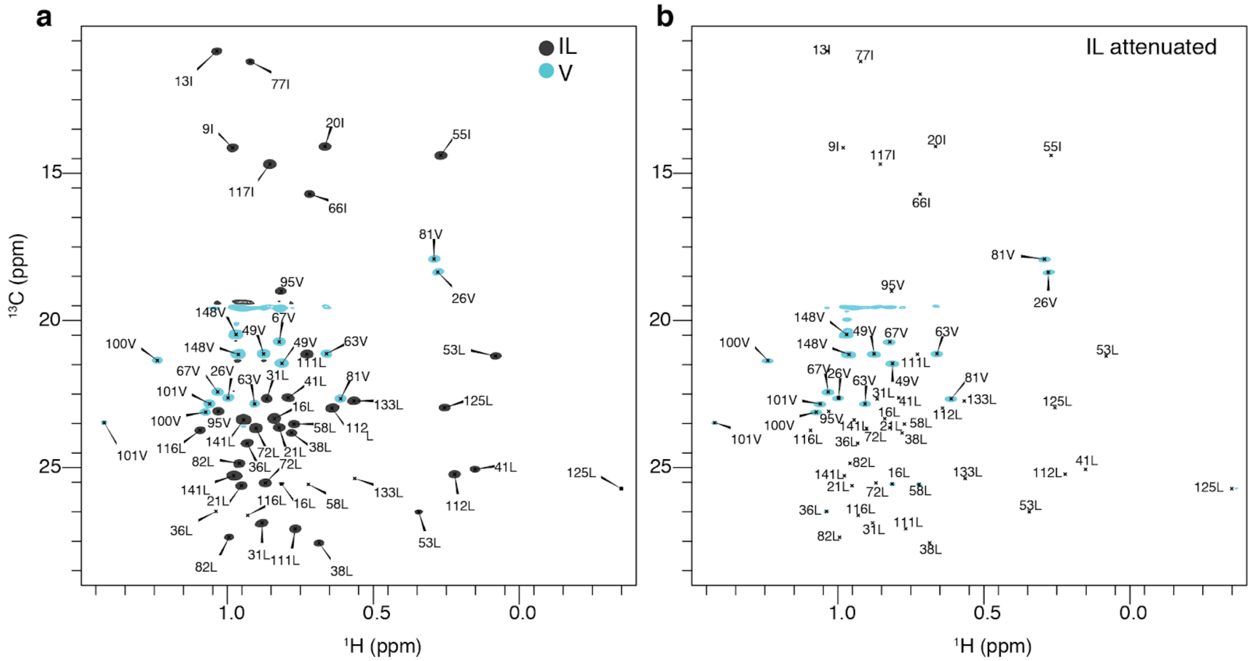

Supplementary Figure 6. Identification of residue types for the methyls of HNH domain. Phase-sensitive 2D ^{1}H-^{13}C constant-time HMQC experiments recorded on ILV* labeled HNH domain using selective decoupling pulse along with constant-time duration of **a**) 28 ms and **b**) 42 ms. Val γ_1/γ_2 methyl signals are positive (colored in cyan).

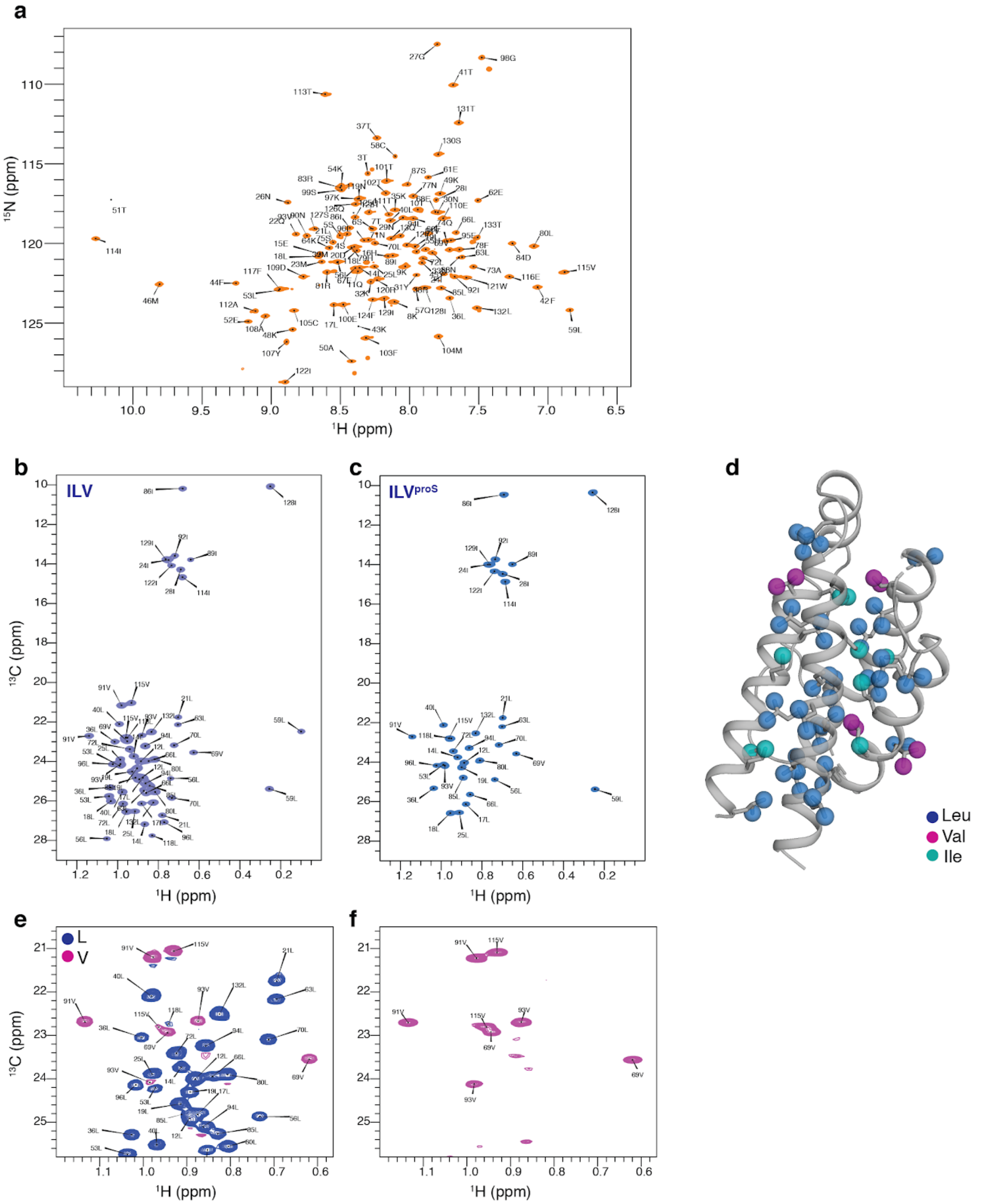

Supplementary Figure 7. Backbone and methyl assignments of interleukin-2. (a) ^{1}H-^{15}N TROSY HSQC, **(b)** ^{1}H-^{13}C methyl HMQC spectra of [U-^{2}H,^{15}N, Ileδ$_1$-^{13}CH$_3$; Leu, Val-^{13}CH$_3$/^{13}CH$_3$] and **(c)** ^{1}H-^{13}C methyl HMQC spectra of [U-^{2}H,^{15}N, Ileδ$_1$-^{13}CH$_3$, Leu, Val proS]-labeled IL-2. **(d)** Distribution of assigned ILV methyl probes in the IL-2 structure (PDB ID 1m47). **(e)** and **(f)** phase-sensitive 2D ^{1}H-^{13}C constant-time HMQC experiments recorded on ILV* labeled IL-2 using selective decoupling pulse along with constant-time duration of **e**) 28 ms and **f**) 42 ms. Val γ$_1$/γ$_2$ methyl signals are positive (colored in purple). All experiments were recorded at ^{1}H field of 800 MHz at 25 ºC, in a 20 mM phosphate buffer, 50 mM NaCl, pH 6.0. Backbone and methyl resonances were assigned as described in **Supplementary Results**.

SUPPLEMENTARY TABLES

Supplementary Table 1: Performance of MAUS and MAGMA on user-defined NOE connectivities for 8 targets in the MAGMA benchmark target set

Target	MW (kDa)	Nº of labeled/ assignable methyls	Labeling scheme	NOE network edges (total/used)[&]	% unique assignments (MAGMA)[%]	% of unique assignments (MAUS)[%]	Time MAGMA (s)	Time MAUS (s)[#]
Ubiquitin	8.6	20/18	ILV	27/27	80	80	0.05	0.7
Ubiquitin (SS*)	8.6	13/10	LV	18/18	77	77	0.02	0.4
MsrB	16.6	22/21	ILV	37/37	77	77	0.02	0.6
EIN1	27	100/84	AILV	145/144	-	43	-	13
ATCase R2	30	39/34	ILV	91/83	41	41	0.8	1.6
MBP	43.4	73/70	ILV	144/140	78	78	2.8	5.4
MSG	81.4	159/141	ILV	230/229	-	40	-	79.5
a7a7	360	56/53	ILV	154/137	93	93	0.1	3.1

*SS - Solid state.

[#] - Time taken to solve subgraph isomorphism problem.

[%] - % unique assignments are calculated as a fraction of respective no. of unique assignments for assignable methyls to labeled methyls.

[&] - Erroneous NOE connectivities were eliminated such that the data graph is subgraph isomorphic with the PDB structure graph. Number of NOE connectivities used for each target are indicated.

Supplementary Table 2: Data acquisition parameters

Protein Name	Labeling Scheme	Sample Concentration	NS<br>300 ms CCH<br>50 ms CCH	AQ<br>300 ms CCH	AQ<br>50 ms CCH
Hβ_2m	AILV	1.3 mM	8<br>8	18.6 ms F1<br>18.6 ms F2	18.6 ms F1<br>18.6 ms F2
HLA-A01	AILV	0.5 mM	8<br>8	16.9 ms F1<br>16.9 ms F2	16.9 ms F1<br>16.9 ms F2
MBP	MILV^{proS}	0.8 mM	8<br>8	26.1 ms F1<br>26.1 ms F2	16.7 ms F1<br>16.7 ms F2
K54	AILV^{proS}	0.5 mM	8<br>8	17 ms F1<br>17 ms F2	13.8 ms F1<br>13.8 ms F2
HNH	ILV	2 mM	8<br>8	13.2 ms F1<br>13.2 ms F2	10.3 ms F1<br>10.3 ms F2
REC2	ILV	0.5 mM	16<br>8	15.8 ms F1<br>7 ms F2	15.8 ms F1<br>13 ms F2
REC3	ILV	0.7 mM	16<br>8	21.4 ms F1<br>10 ms F2	21.4 ms F1<br>10 ms F2
IL-2	ILV	0.4 mM	8<br>8	27 ms F1<br>12.4 ms F2	12.4 ms F1<br>12.4 ms F2

NS = number of scans
AQ = acquisition time
F1 = Cm (NOE dimension)
F2 = Cm (attached to direct 1H)

Supplementary Table 3: Clustering and Symmetrization possibilities arising from the spectral complexity

Target	Total clustering possibilities	Total symmetrization possibilities	Valid clustering possibilities	Reduced symmetrization possibilities	Total no. of data graphs
Hβ_2m	2^{12}	2^9 x 3	2^2	2^7 x 3	2^9 x 3
HLA-A01	2^{32}	2^{32} x 3^5 x 5 x 7^2	2^3	2^{27} x 3^2	2^{30} x 3^2
HLA-A02	2^{41}	2^{38} x 3^5 x 7	2^2	2^{31} x 3 x 7	2^{33} x 3 x 7
MBP	2^{30}	2^{11} x 3^2	2^2	2^{10}	2^{12}
IL-2	2^{46} x 3^{12}	2^{20} x 3^4	2^8 x 3^3	2^{16} x 3	2^{24} x 3^4
HNH	1	2^7 x 3^5 x 5	1	2^7 x 3	2^7 x 3
REC2	2^{35} x 3	2^7 x 3	2 x 41	2^3	2^4 x 41
REC3	2^3	2^{29} x 3 x 5^2	2	2^{22} x 3 x 5	2^{23} x 3 x 5

Supplementary Table 4: MAUS results using a residue type classifier for Leu/Val methyl peaks

Target	% unique assignments	% options > 1 and <= 3
Hβ_2m	89	11
HLA-A01	60	32
HLA-A02	60	11
MBP	71	17
HNH	89	11
IL-2	36	39
REC2	54	17
REC3	49	36

For each target, the residue types of Leucine and Valine methyl peaks in the 2D input list were replaced with the ambiguous LV annotation, and an automated classifier within MAUS was used to provide 99% confident residue type classifications from their chemical shift values. LV peaks which could not be confidently classified, remained ambiguous during the subsequent MAUS resonance assignment enumeration steps. To invoke the residue type classifier, the user can select “Auto” for the “Peak residue type assignment” field in the input form.

Supplementary Table 5: Comparison of MAUS with existing methyl assignment methods

Method	Availability (academic users)	May output erroneous assignments	Provides >50% unambiguous assignments	Runtime in minutes (worst case)	Runs directly on 3D/4D NOE peak data
MethylFLYA	Commercial	Yes	No	~2,553	Yes
MAGMA	Free	No	Yes	Time out[&]	No
MAGIC	Free	Yes	No	Time out*	Yes
FLAMEnGO2.4	Free	Yes	No	Time out*	Yes
MAP-XSII	Free	Yes	No	~16	Yes
MAUS	Free (Web-server)	No	Yes	~16[%]	Yes

[&] After 167 min of runtime (MAGMA limit)
* After 96 hr of runtime
[%] Includes time taken by MAUS to run the Rosetta side chain optimization protocol, evaluate all possible data graphs consistent with the data, and exhaustively enumerate all assignment solutions

Supplementary Table 6: Sequences of codon optimized genes for *E. coli* expression

Protein	Codon optimized genes
**Cas9 HNH domain**	aacagccgtgagcgtatgaagcgtatcgaggaaggtattaaagaactgggcagccagattctgaaggagcacccggtg<br>gaaaacacccagctgcaaaacgagaaactgtacctgtactatctgcagaacggtcgtgatatgtatgttgaccaagaact<br>ggatatcaaccgtctgagcgactacgatgtggaccacattgttccgcaaagcttcctgaaggacgatagcatcgacaaca<br>aagtgctgacccgtagcgacaaaaaccgtggcaagagcgataacgttccgagcgaggaagtggttaagaaaatgaag<br>aactattggcgtcagctgctgaacgcgaaactgatcacccagcgtaagtttgataacctgaccaaggcggaacgtggtg<br>gcctgggtggcggtagcggtacccgtctgccgaagaaaaagcgtaaagtgggcggtggcagccaccaccaccacca<br>ccac
**Cas9 REC2 domain**	acatttcctgatcgagggcgacctgaacccggacaatagcgacgtggacaaactgttcattcaactggtgcagacctaca<br>atcaactgttcgaggaaaacccgatcaacgcgagcggtgtggatgcgaaggcgattctgagcgcgcgtctgagcaaaa<br>gccgtcgtctggagaacctgatcgcgcagctgccgggtgaaaagaaaaacggtctgtttggcaacctgattgcgctgag<br>cctgggcctgaccccgaacttcaagagcaactttgatctggcggaggacgcgaagctgcaactgagcaaagatacctа<br>cgacgatgacctggacaacctgctggcgcagatcggcgatcaatatgcggacctgttcctggcggcgaaaaacctgag<br>cgatgcgattctgctgagcgacattctgcgt
**Human IL-2**	atggccccgacaagttcaagcacaaagaagacccagttacaattagagcatcttctgctgganttgcagatgatcttaaac<br>ggtattaataattacaaaaatcccaagttgactcgtatgctgacgtttaaattctatatgccaaaaaaggctacggagcttaa<br>acatctgcaatgcctggaagaggagcttaaaccgttggaggaagttcttaatcttgcccagagtaagaatttccacttacgt<br>ccgcgtgacttgattagtantatcaatgtgatcgtattggaattgaaaggcagtgagacgacgttcatgtgcgaatatgcag<br>acganacggcgaccatcgtggagttcctgaatcgctggattacgttctcccaatccatcatctccactcttacc

Supplementary Table 7: Input parameters and NOE statistics

Target	Total NOEs	Diagonal NOEs	Number of symmetry matches	Geminal cluster	NOEs with SC size# = 2	NOEs with SC size = 3	NOEs in CC&	NOEs used	EDC*
$H\beta_2m$	196	35	24	28	84	21	4	105	3.1
HNH	232	1	46	46	114	21	4	135	2.8
HLA-A01	510	95	38	62	252	9	54	261	3.5
HLA-A02	533	98	34	76	244	21	60	265	3.1
MBP	434	76	58	0	272	24	4	296	3.9
IL-2	347	48	8	59	186	30	16	216	3.9
REC2	250	0	25	58	158	9	0	167	3.7
REC3	487	88	12	77	238	57	15	295	3.5

* - EDC or effective degree connectivity is calculated as (2* (no. of edges of the data graph)/ no. of nodes).
#SC - simple components
&CC - complex components

Supplementary Table 8: Performance of MAUS using 8 Å 50ms NOE distance threshold

Target	% unique assignments	% options <= 3 and > 1
Hβ_2m	77	23
HLA-A01	64	30
HLA-A02	65	17
MBP	61	29
IL-2	26	31
HNH	89	11
REC2	33	12
REC3	67	28

SUPPLEMENTARY RESULTS

Comparison with MAGMA: We compared MAUS with the graph theory-based approach, MAGMA. Specifically, we carried out exhaustive subgraph isomorphism computations using SAT in MAUS and VF2 in MAGMA (v1.2.3). As a first test set, we used previously published connectivities derived from a manual analysis or raw NOE peaks for 8 protein targets of varying sizes and complexities, included in the MAGMA benchmark, to define input data graphs, *H,* and we used MAGMA's method for constructing the graphs *G* from the PDB structure of each target. Out of 8 targets, 5 included a small number of erroneous (false positive) NOE connectivities, due to user error. To evaluate the performance of the two methods in solving the sub-graph isomorphism problem using real data, we removed these erroneous edges and then performed calculations with both VF2 and SAT on the same inputs (**Supplementary Table 1**). While SAT solves smaller cases in sub-seconds time, providing the same assignment outputs as VF2, for larger, more complex graphs in the MAGMA benchmark (>80 connected methyls, EIN and MSG) SAT maintains a robust performance while the VF2 algorithm times out after 10,000 seconds. MAGMA's VF2 algorithm is exhaustive, however, it is limited to targets of smaller to moderate size. It should be emphasized that MAGMA uses an alternative strategy and algorithm to address erroneous connectivities that may arise from manually processing of the NOE data, while MAUS *eliminates* erroneous NOE connectivities using a massive amount of milliseconds-timescale SAT computations, operating *directly* on the unprocessed 3D NOE data.

To test this trend further, we carried out a detailed performance comparison of VF2 and SAT using simulated data from a non-redundant set of 147 PDB structures. For each PDB structure graph, we simulated 10 isomorphic data sub-graphs by removing edges until we reached a desired sparsity level (represented by an effective degree connectivity, or EDC, calculated as (2*no. of the edges)/(no. of nodes in the data graph)). We found that MAUS is 1,000X faster for 536 out of 3,275 simulations (**Supplementary Fig. 1**). From these results, it becomes evident that the special-purpose SAT algorithm is highly robust for graphs representative of complex protein folds, in contrast to VF2.

Strategy for the assignment of Ile-δ_1, Leu and Val methyl correlations in the Cas9 domains: The backbone and methyl assignment of the Cas9 domains were obtained by following a divide-and-conquer approach in which the large system is dissected into smaller building blocks (HNH, REC2 and REC3 domains) using standard 'backbone' and 'side-chain' triple resonance multidimensional NMR methods. *De novo* stereospecific methyl assignments were achieved by utilizing three independently prepared isotopically labeled samples: ILV (Ile $^{13}C\delta_1$; Leu $^{13}C\delta_1/^{13}C\delta_2$; Val $^{13}C\gamma_1/^{13}C\gamma_2$ in an otherwise U-[^{15}N, ^{12}C, ^{2}H] background), ILV^{proS} (Ile $^{13}C\delta_1$; Leu $^{13}C\delta_2$; Val $^{13}C\gamma_2$ in an otherwise U-[^{15}N, ^{12}C, ^{2}H] background) and ILV* (Ile $^{13}C\delta_1$ only; Leu $^{13}C\delta_1/^{13}C\delta_2$; Val $^{13}C\gamma_1/^{13}C\gamma_2$ in an otherwise U-[^{15}N, ^{13}C, ^{2}H] background). We employed a multipronged approach where backbone assignments are used to aid the assignment of side-chain methyl groups. Specifically, we first obtained backbone assignments using TROSY-based 3D HNCA, HN(CA)CB and HNCO experiments recorded with the ILV* labeled samples. Final backbone assignments were further validated using TALOS-N. Next, Ile, Leu and Val methyl assignments were achieved using methyl-to-amide NOEs obtained from 3D H_N-C_MH_M SOFAST NOESY and 3D N-C_MH_M SOFAST NOESY experiments. Final methyl assignments were cross-

validated using methyl-to-methyl NOEs obtained from 3D H_M-C_MH_M SOFAST NOESY and 3D C_M-C_MH_M SOFAST NOESY experiments. Leu/Val geminal pairs were determined by comparing NOE strips in 3D C_M-C_MH_M SOFAST NOESY experiments recorded using either short (50 ms) and long (300 ms) mixing times. Finally, stereospecific Leu δ_2 and Val γ_2 methyl assignments were achieved by comparison of 2D ^1H-^{13}C HMQC spectra of ILV and ILV^{proS} labeled samples. The reference assignments cover a large fraction (>90%) of all the methyls groups of the blind targets.

For REC2, three leucine methyls (L169, L195 and L301) are spatially located at the protein surface and only the geminal pairing were observed in the 3D C_M-C_MH_M SOFAST NOESY (300 ms mixing time). Due to the absence of amide to methyl and longer methyl-methyl NOE restraints these methyl groups could not be assigned unambiguously. When ambiguity cannot be resolved based on high confidence score values, MAUS will output multiple alternative assignments. A final assignment completeness of 91% for ILV sidechain methyls, is reported in the BMRB entry 28105.

For REC3 domain, the ^1H-^{15}N transverse relaxation optimized spectroscopy (TROSY) spectrum was of marginal quality (**Supplementary Fig. 5d**) with a significant fraction of completely broadened amide resonances (~55%, residues 660-712). These are mainly residues spatially close to the RuvC domain in the Cas9-sgRNA-DNA complex and could have critical role on the conformational dynamics of the REC lobe of Cas9, where allosteric function relies on the opening of the REC3 region to integrate the incoming RNA:DNA hybrid. Most of the detected but unassigned spin systems show low signal intensity, lack sequential contacts and NOE connections, or are in overlapping regions, thereby precluding unambiguous assignment. About 45% of the backbone assignments of REC3 (21/49 ILV methyls) were cross-validated through methyl-to-amide NOEs from 3D H_N-C_MH_M and 3D N-C_MH_M SOFAST NOESY experiments. Iterative completion (28 ILV methyls) and cross-validation of the methyl assignments were achieved through NOEs obtained from 3D H_M-C_MH_M and 3D C_M-C_MH_M SOFAST NOESY experiments allowing full assignment of the ILV methyl resonances. A final assignment completeness of 45% and 100% for non-Pro backbone amides and ILV sidechain methyls, respectively, is reported in the BMRB entry 28110.

Assignment of Ile-δ_1, Leu and Val methyl correlations in human IL-2: The backbone assignments of human IL-2 was previously published (BMRB 6621), however due to differences in buffer and pH condition in the present study (20 mM sodium phosphate, 50 mM sodium chloride, pH 6.0), we determined *de novo* assignments for IL-2. Backbone amide and side-chain methyl assignments were cross-validated using methyl-to-amide NOEs obtained from 3D H_N-C_MH_M and N-C_MH_M SOFAST NOESY and validated using methyl-to-methyl NOEs obtained from 3D H_M-C_MH_M and 3D C_M-C_MH_M SOFAST NOESY experiments, as described above. Val and Leu amino-acid type were unambiguously distinguished using a specially designed selective homonuclear decoupling pulse during the indirect ^{13}C chemical shift encoding delay recorded on ILV* labeled[2] (**Supplementary Fig. 7**). A final assignment completeness of 100% for non-Pro backbone amides and ILV sidechain methyls, is reported in the BMRB entry 28104.

SUPPLEMENTARY REFERENCES

1. Cordella, L. P., Foggia, P., Sansone, C. & Vento, M. A (sub)graph isomorphism algorithm for matching large graphs. *IEEE Trans. Pattern Anal. Mach. Intell.* **26**, 1367–1372 (2004).
2. Behera, S. P. *et al.* Nearest-neighbor NMR spectroscopy: categorizing spectral peaks by their adjacent nuclei. *Nat. Commun.* **11**, 5547 (2020).
