# Supplementary material for: Backbone-independent NMR resonance assignments of methyl probes in large proteins
Source: Nat Commun. 2021 Jan 29;12:691. doi: 10.1038/s41467-021-20984-0 (PMC7846771; doi:10.1038/s41467-021-20984-0)
Supplement: Supplementary file 3 — Reporting Summary [file 41467_2021_20984_MOESM3_ESM.pdf]

nature research

Corresponding author(s): Nikolaos G. Sgourakis

Last updated by author(s): December 1st, 2020

Reporting Summary

Nature Research wishes to improve the reproducibility of the work that we publish. This form provides structure for consistency and transparency in reporting. For further information on Nature Research policies, see our Editorial Policies and the Editorial Policy Checklist.

Statistics

For all statistical analyses, confirm that the following items are present in the figure legend, table legend, main text, or Methods section.

n/a	Confirmed	
☐	☒	The exact sample size (n) for each experimental group/condition, given as a discrete number and unit of measurement
☐	☒	A statement on whether measurements were taken from distinct samples or whether the same sample was measured repeatedly
☒	☐	The statistical test(s) used AND whether they are one- or two-sided<br>*Only common tests should be described solely by name; describe more complex techniques in the Methods section.*
☒	☐	A description of all covariates tested
☒	☐	A description of any assumptions or corrections, such as tests of normality and adjustment for multiple comparisons
☒	☐	A full description of the statistical parameters including central tendency (e.g. means) or other basic estimates (e.g. regression coefficient) AND variation (e.g. standard deviation) or associated estimates of uncertainty (e.g. confidence intervals)
☒	☐	For null hypothesis testing, the test statistic (e.g. F, t, r) with confidence intervals, effect sizes, degrees of freedom and P value noted<br>*Give P values as exact values whenever suitable.*
☒	☐	For Bayesian analysis, information on the choice of priors and Markov chain Monte Carlo settings
☒	☐	For hierarchical and complex designs, identification of the appropriate level for tests and full reporting of outcomes
☒	☐	Estimates of effect sizes (e.g. Cohen's d, Pearson's r), indicating how they were calculated

Our web collection on statistics for biologists contains articles on many of the points above.

Software and code

Policy information about availability of computer code

Data collection	Topspin 3.5pl7 (Bruker)
Data analysis	Analysis of NMR data was performed using NMRPipe (Version 10.9 Rev 2020.119.13.27, Frank DeLaglio, NIST), NMRFAM SPARKY (Version 1.3, Tom Goddard and Tom Ferrin), CCpNmr (Version 2.4.2, CCPN), ExPASy ProtParam (Gasteiger, et.al.) . All these softwares are available in the public domain, and all relevant parameters used to analyze the data are outlined in detail in the Methods section of the article.<br>A web-server running the MAUS software is available at https://methylassignment.chemistry.ucsc.edu

For manuscripts utilizing custom algorithms or software that are central to the research but not yet described in published literature, software must be made available to editors and reviewers. We strongly encourage code deposition in a community repository (e.g. GitHub). See the Nature Research guidelines for submitting code & software for further information.

Data

Policy information about availability of data

All manuscripts must include a data availability statement. This statement should provide the following information, where applicable:

- Accession codes, unique identifiers, or web links for publicly available datasets
- A list of figures that have associated raw data
- A description of any restrictions on data availability

Nuclear Magnetic Resonance assignments for IL-2, REC2, HNH and REC3, used as blind targets have been deposited in the BMRB under accession numbers 28104, 28105, 28106, and 28110 respectively.
The unprocessed and processed NMR data along with the structural models and input parameters used to run MAUS for all targets in this study are available at https://methylassignment.chemistry.ucsc.edu

Field-specific reporting

Please select the one below that is the best fit for your research. If you are not sure, read the appropriate sections before making your selection.

- [x] Life sciences
- [ ] Behavioural & social sciences
- [ ] Ecological, evolutionary & environmental sciences

For a reference copy of the document with all sections, see nature.com/documents/nr-reporting-summary-flat.pdf

Life sciences study design

All studies must disclose on these points even when the disclosure is negative.

Sample size	Sample size is not applicable to NMR measurements described in the manuscript since our NMR experimental results do not report and do not depend on quantitative values.
Data exclusions	No data was excluded in this study.
Replication	Data replication does not apply to NMR measurements described in this manuscript. Standard referencing of chemical shifts is applied to ensure consistency and reproducibility.
Randomization	Randomization does not apply to NMR measurements described in this manuscript since there are no head-head comparisons between cohorts.
Blinding	Blinding is not relevant since the study does not compare different groups of measurements.

Reporting for specific materials, systems and methods

We require information from authors about some types of materials, experimental systems and methods used in many studies. Here, indicate whether each material, system or method listed is relevant to your study. If you are not sure if a list item applies to your research, read the appropriate section before selecting a response.

Materials & experimental systems

n/a	Involved in the study	
☒	☐	Antibodies
☒	☐	Eukaryotic cell lines
☒	☐	Palaeontology and archaeology
☒	☐	Animals and other organisms
☒	☐	Human research participants
☒	☐	Clinical data
☒	☐	Dual use research of concern

Methods

n/a	Involved in the study	
☒	☐	ChIP-seq
☒	☐	Flow cytometry
☒	☐	MRI-based neuroimaging
